# Infection due to *Mycoplasma hominis* after left hip replacement: case report and literature review

**DOI:** 10.1186/s12879-019-3686-z

**Published:** 2019-01-14

**Authors:** Lili Xiang, Binghuai Lu

**Affiliations:** 1 Department of Laboratory Medicine, Chongqing Shapingba District Chenjiaqiao hospital, Chongqing, China; 2 Affiliated Hospital of Chongqing Medical and Pharmaceutical College, Chongqing, China; 3 Laboratory of Clinical Microbiology and Infectious Diseases, Department of Pulmonary and Critical Care Medicine, China-Japan Friendship Hospital, Beijing, China; 4 Center for Respiratory Diseases, China-Japan Friendship Hospital, Beijing, China; 5 National Clinical Research Center of Respiratory Diseases, Beijing, China

**Keywords:** *Mycoplasma hominis*, Postoperative infections, Hip replacement

## Abstract

**Background:**

Hip replacement is generally conducted in those with prolonged arthritis pain or hip fractures, and postoperative infection is a serious complication. *Mycoplasma hominis*, belonging to mycoplasma species, exists mainly in the genitourinary tract. *M. hominis* infection after total hip replacement was rarely documented in literature.

**Case presentation:**

A 59-year-old male was febrile after left total hip replacement. Empiric therapy with cefepime for suspected infection was ineffective. Specimens at the infection site were collected for culture, and pinpoint colonies grew after incubation at 35 °C for 48 h on blood agar plate. They grew to approximately 0.5 mm colonies in diameter after 7-day incubation, and were identified as *M. hominis*. Sequentially, combination therapy with clindamycin hydrochloride and moxifloxacin was initiated, and the patient defervesced within 3 days and was discharged home.

**Conclusions:**

The study highlighted the potential pathogenicity of *M. hominis* in postoperative infection. The possibility of this microorganism involvement should be valued if the patients who experienced the hip or joint replacement had inexplicable fever.

## Background

*Mycoplasma hominis* is a commensal bacterium of the urogenital tract and generally responsible for pelvic inflammatory illnesses and postpartum and neonatal infections [1–3]. *M. hominis* infections outside the genitourinary tract occurred rarely. However, to date, wound infection [4], meningitis [5], postoperative infections [6–9] and other disseminated infections in immunocompromised patients [10–12] due to the organism have been increasingly documented. Furthermore, hip replacement is commonly-conducted surgery to relieve obstinate arthritis pain or fractures in China. Herein, a case of extragenital infection caused by *M. hominis* after hip replacement was reported. Furthermore, we reviewed relevant literature to highlight the potential pathogenicity of *M. hominis* in postoperative infection.

## Case presentation

### Medical history

**Fig. 1:**
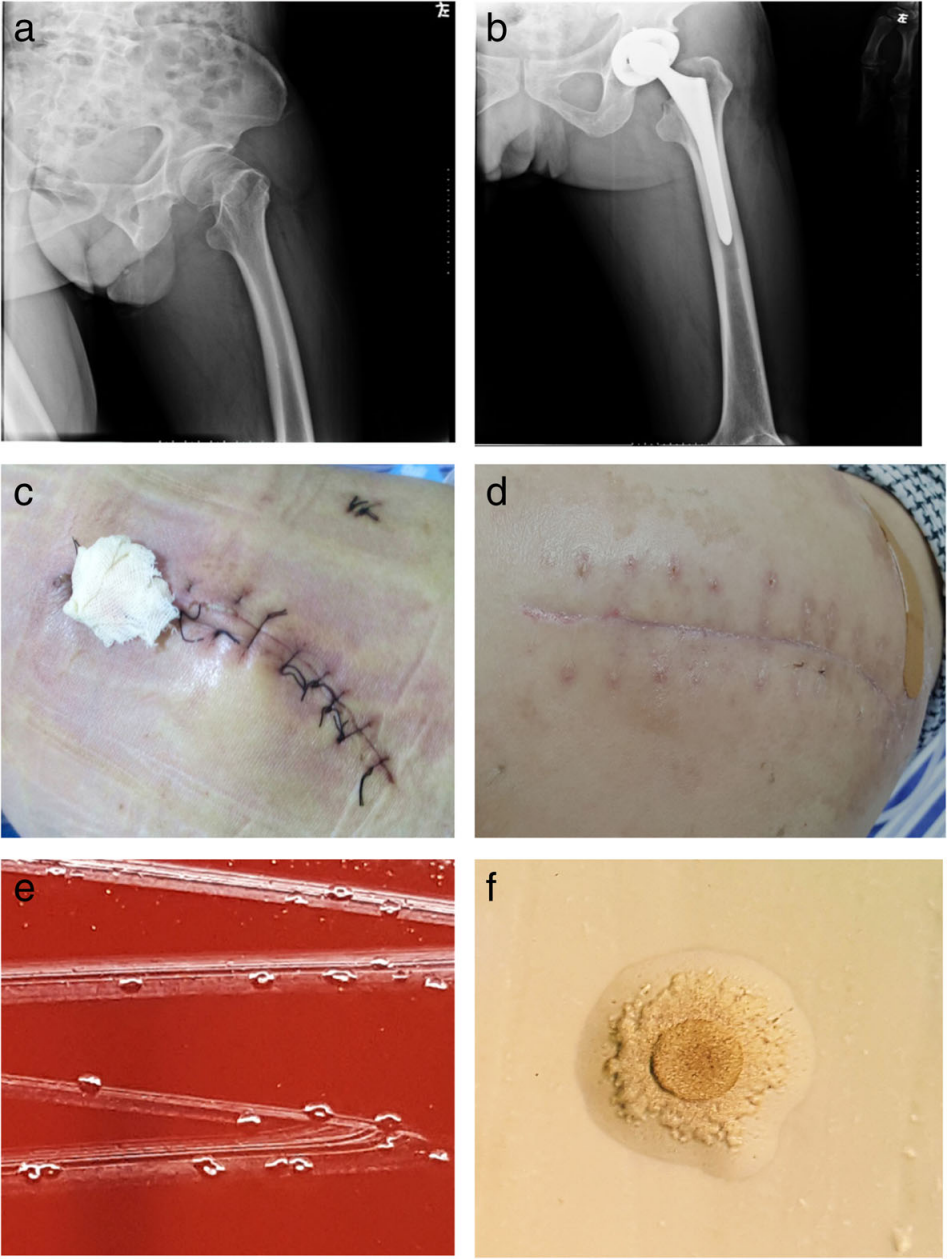
**a** Fractures of left femoral neck under X-ray at admission. **b** Prosthetic hip in right position (2 h after hip replacement under X-ray). **c** Redness and swelling around surgical site, wound fluctuation in palpation (8 days after surgery). **d** Recovered after anti- *M. hominis* treatment with the combination of moxifloxacin, and doxycycline (18 days after surgery). **e** After 7 day of incubation of subcutaneous fluid collected during debridement on blood agar at 37 °C in a 5% CO_2_ atmosphere, tiny, nonhemolytic, transparent colonies grew on Columbia blood agar plate. **f** Fried-egg-type colonies of *Mycoplasma hominis* on solid media 5 days after subculture

**Fig. 2:**
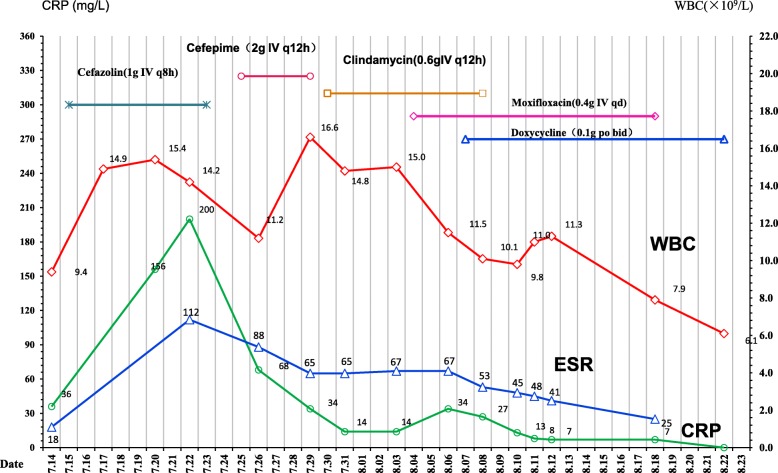
Relationship between the change of inflammatory markers (WBC, ESR and CRP) and the use of antibiotics during hospitalization. PMN: Polymorphonuclear leucocyte; ESR: erythrocyte sedimentation rate; WBC: White blood cells

A 59-year-old male was admitted to Chongqing Shapingba District Chenjiaqiao hospital, Chongqing, China. He suffered the fractures of left femoral neck after falling to the ground (Fig. 1a). On July 16, 2017, the left total hip replacement was conducted and prosthetic hip in position was shown under X-ray (Fig. 1b). Cefazolin sodium (1 g IV q8h) was started for prophylactic administration. His indwelling urinary catheter was removed after 24 h. On the 8th day after surgery, however, the patient presented with left hip pain and clinical signs of infection, including fever (38.5 °C), redness and swelling around the surgical site (Fig. 1c), and he also reported local tenderness. His blood examination demonstrated the white blood cell (WBC), C-reactive protein (CRP), and erythrocyte sedimentation rate (ESR) significantly increased during postoperative period, as shown in Fig. 2. Furthermore, the screening tests for human immunodeficiency virus, hepatitis B virus and hepatitis C virus infections were non-reactive, and no abnormality in liver or renal function tests was observed. His T-lymphocyte subsets and gamma-globulin analysis were within normal range.

On July 25, 2017, approximately 400-ml light-yellow, odorless subcutaneous fluid was punctured at surgical site, and forwarded to the microbiological laboratory for bacterial smear and culture. A large amount of polymorphonuclear leucocytes (PMNs) were detected but no microorganism on gram-staining smear. Moreover, there was a negative growth on the blood and chocolate agar plates. The repeated blood cultures using the BacT/ALERT 3D blood culture microbial detection system (bioMérieux SA, Marcy l’Étoile, France) were negative. The post-surgical infection was still under suspicion, and the wound was cleaned with iodophor and drainage gauze was placed. Cefepime (2 g IV q12h) was administrated. However, empiric therapy was still ineffective, the prosthetic hip infection deteriorated, fever persisted, and on July 26, the debridement of left prosthetic hip was performed and the seroma, superficial fascia, deep fascia, deep tissue, as well as subcutaneous fluid collected during the surgery were sent for culture on blood and chocolate agar plates. Unexpectedly, tiny colonies grew after 48 h, and they grew to approximately 0.5 mm colonies in diameter after 7-day incubation (Fig. 1e), and were identified as *M. hominis* by the bioMérieux® SA Mycoplasma IST2 kit (bioMérieux, France). The identification was also confirmed with 16S rRNA sequencing.

In vitro antimicrobial susceptibility testing (AST) revealed that the organism was susceptible to doxycycline, clindamycin and levofloxacin, but resistant to azithromycin by the bioMérieux® SA Mycoplasma IST2 kit (Biomerieux, France). Combination therapy with clindamycin hydrochloride (0.6 g IV q12h) and moxifloxacin (400 mg IV QD) was initiated, as shown in Fig. 2. The patient defervesced within 3 days. His infection site recovered gradually (Fig. 1d). Repeated X-ray scans before discharge showed marked improvement of his prosthetic hip. The patient was discharged on August 23, without further complications. No recurrence of symptoms and signs was reported during three-month outpatient follow-up.

### Microbiological test

After 48 h of incubation on blood agar at 37 °C in a 5% CO_2_ atmosphere, pinpoint-sized, non-hemolytic, and transparent colonies were found on Columbia blood agar plate. The colonies were difficult to emulsify in saline water during the preparation of suspension solution for identification and AST. Both Gram stain and Wright-Giemsa mixed stain of the isolates demonstrated no bacterial morphology under × 1000 magnification, and only granular aggregates were detected. Fried-egg-type colonies of *M. hominis* growed on solid media (Zhongqisheng Hebei Bio-tech Co., Ltd.) 5 days after subculture (Fig. 1f).

## Discussion and conclusions

*M. hominis* is part of the normal inhabitant of the genitourinary tract [6]. However, in line with publicly-available documents, it might disseminate to other body sites secondary to a disruption of the mucosa or in patients with autoimmune disorders, hypogammaglobulinemia, and other underlying immunosuppressions [3, 12–15]. Herein, we described the clinical circumstances, treatment, and outcomes of a postoperative septic complication due to the microorganism after hip replacement. To the best of our knowledge, this is the first report of *M. hominis* as the causative, fastidious agent of prosthetic hip infection in China.

To better understand the characteristics of postoperative infection after hip or knee replacement, PubMed was searched for literature review and 5 cases by *M. hominis* in 4 reports were included for comparison (Table 1) [16–19]. The literature review demonstrated that, including our case, the gender ratio of male/female suffered *M. hominis* infection after joint or tip replacement was 5:1. The median age was 64 years old. Furthermore, as documented, CRP concentrations were available in 4 out of 5 cases, and all their CRP levels were higher than 100 mg/L. Our case also had an increased CRP (200 mg/L), hinting elevated CRP level would help in suspecting postoperative *M. hominis* infection. The review also described that the most common and effective test for diagnosing joint or hip *M. hominis* infection was the culture of wound exudation, joint fluid, and aspiration fluid of the knee [16–19]. In our case, *M. hominis* was recovered from subcutaneous puncture fluid and successfully identified. This showed that, if the tiny colonies grew on the blood agar plate without obvious bacteria shapes under gram-staining smears, *M. hominis* should be suspected of being underlying pathogen [16, 17]. Presently, the molecular methods, such as 16S rRNA sequencing or real-time PCR, might be used for the identification of infections caused by the bacterium [17, 19].

**Table 1:** Summary of the reported cases of *Mycoplasma hominis* after joint or hip replacement

	1 [16]	2 [17]	3 [17]	4 [18]	5 [19]	6 current case
Case Number	1	1	1	1	1	1
Age (years)	62	71	76	66	54	59
Surgery	Left total knee replacement due to knee osteoarthritis.	Left total knee replacement.	Left knee joint replacement.	Bilateral total knee replacements 5 years ago and a left total hip replacement 2 years ago.	Implantation of a total hip prosthesis one month before.	Left hip replacement.
Gender	Male	Male	Male	Female	Male	Male
Fever	Yes	39.7 °C	38.7 °C	Yes	Yes	38.5 °C
Infection indicators						
CRP(mg/L)	208.3	122.4	143.6	NA	374	200
WBC(× 10^9^/L)	NA	10.25 (Neutrophils 81.4%)	11.83 (Neutrophils 88.3%)	6.0	6.9	15.4
ESR(mm/hr)	NA	61	63	101	NA	112
Microbiological test results	Bacterial and fungi culture of wound exudation and seepage demonstrated a negative growth, whereas the secretion collected in the operation suggested a positive *M. hominis* growth, identified by mass spectrometer.	Pinpoint, translucent colonies on Brucella agar after 2-day incubation of joint fluid, confirmed as *M. hominis* by 16S rRNA sequencing.	Anaerobic culture for 3 days incubation of joint fluid indicated the growth of *M. hominis*.	Cultures of the aspiration fluid of the knee revealed very small clear colonies were seen on the blood agar plates, present on both the aerobic and anaerobic plates.	At the time of admission, Gram stain of a swab taken from the wound of the right hip showed rare leukocytes but no bacteria, and cultures were negative. Cultures of the effusion collected via arthroscopy of the left knee remained negative. The biopsy of the inflamed tissue revealed no bacteria on Gram staining, and no growth after 14-day culture. *M. hominis*. Was identified *via*16S rRNA sequencing.	After 48 h of incubation on blood agar at 37 °C in a 5% CO_2_ atmosphere, tiny, non-hemolytic, transparent colonies were found on Columbia blood agar plates.
Presentation of post-surgical infection	Blood seeping and pale clear liquid exudation from the wound were observed on the 3rd and 4th day after the surgery.	Three days after operation, there were redness and swelling, pain at surgical site.	One day after operation, fever, redness and swelling around knee were observed.	NA	Implantation of a total hip prosthesis one month before. Symptoms of a septic arthritis in both knees and hips and delayed wound healing and fistula formation after implantation of a total hip prosthesis one month before.	Eight day after surgery, the patients presented with left hip pain and clinical signs of infection, including fever (38.5 °C), redness and swelling around the surgical site. And he reported significant local press pain. Approximately 400-ml light yellow, odorless effusion of the wound was drained.
Antibiotic prevention	Cefazolin	Ceftazidime, vancomycin	Vancomycin	NA	NA	Cefazolin
Antibiotic treatment.	Cefazolin was replaced by vancomycin. Later transferred to the combination into erythromycin, clindamycin and minocyline.	Vancomycin, metronidazole.	Vancomycin. Later switched to azithromycin, doxycycline, moxifloxacin.	Cefazolin 500 mg of tetracycline iv every 8 h. After the first week, switched to oral doxycycline (200 mg/day), and over the next 3 weeks.	Ciprofloxacin and clindamycin; subsequently changed to cefazolin and clindamycin, continued for 4 weeks; and later changed to moxifloxacin and rifampin for a presumed chronic *S. epidermidis* infection. Treatment with moxifloxacin was initiated, however the patient’s condition continued to deteriorate.	Cefepime, clindamycin, moxifloxacin, and doxycycline.
Blood culture	Negative	Negative	Negative	Negative	Negative	Negative
Outcome	Recovery	Recovery	Recovery	Recovery	Dead	Recovery

*WBC* white blood cell, *CSF* cerebrospinal fluid, *N* negative, *NA* not available, *w* week, *y* year

Furthermore, it is difficult to clarify the possible portal of entry of *M. hominis* in cases of this postoperative infection [20]. In accordance with previous reports, the source for an *M. hominis* in postoperative hip or joint infections might be explained by seeding of surgical site through transient bacteraemia. This bloodstream infection occurred after urinary catheterization if the genitourinary tract had been colonized by the microorganism. Indeed, urinary tract catheterization has been associated with *mycoplasma* bacteraemia leading to the seeding of brain-damaged tissues in brain abscess cases [5, 6, 9, 21]. Similarly, in our case, a possible pathway might be indwelling catheter used during surgery and a possible route for hematogenous spread to surgical site. However, it is rather difficult to definitively identify the source of infection.

It presented a challenge to identify *M. hominis* as pathogen due to its elusiveness and fastidious slow-growing nature [5, 8, 13, 22, 23]. This might be explained by the following reasons. Firstly, *M. hominis* has a 3-layer sterol membrane but lacks cell wall. Consequently, the inability to detect *Mycoplasma spp.* by routine gram-staining contributes to the failure of detection in the clinical specimens [24]. In present case, the gram-staining and Wright-Giemsa mixed staining smear of subcutaneous fluid and the colonies demonstrated no bacterial morphology. Secondly, the slow-growth properties of *M. hominis* made the detection on plates challenging, because it generally takes several days (often ≥2 days) to grow into tiny colonies on the media commonly used in laboratory and the requirement for extended incubation period makes a timely diagnosis less likely. And moreover, the routine biochemical methods might fail to identify it correctly [18]. Thirdly, it is rather difficult to detect the growth of *M. hominis* in standard blood culture bottle solutions that use polyanethol sulfonate as an anticoagulant but rather requiring special methods for growth through automatic detection systems in those with suspected bacteremia, and false-negative results are likely yielded [16–19, 24]. Taken together, the post-surgical *M. hominis* infection cases are not readily detected via standard microbiology methods [7, 19]. Considering the high urethral carriage rate of *M. hominis* (~ 15% of healthy adults) and catheterization is a common procedure during operation, the possibility of postoperative *Mycoplasma* infection might be under-diagnosed or reported [8].

Our Pubmed review showed that 83.3% (5/6) of the patients survived after appropriate antimicrobial treatment. Furthermore, previous studies demonstrated that the mycoplasmas resulted in serious infections without timely detection [15, 19, 22]. For example, a patient with implantation of a total hip prosthesis died due to a postoperative hip prosthesis and disseminated infection by *M. hominis* and *Ureaplasma parvum* [19]. Accordingly, if a patient developed unexplained post-surgical fever in cases of otherwise culture-negative infections, particularly if treat with wide-spectrum antibiotics meets with a poor response, it is especially important to consider the potential of *Mycoplasmas* as pathogens.

Empiric therapy for postoperative infections generally includes agents such as beta-lactams and vancomycin that act on the bacterial cell wall. Such an initial therapy will show no efficacy against *M. hominis* infections due to its lack of cell wall [16, 17]. Furthermore, *M. hominis* is, in contrast to most mycoplasmas, intrinsically resistant to currently available macrolide antibiotics due to the mutations in the 23S rRNA gene and is the only mycoplasma susceptible to clindamycin, which often used for eradicating *M. hominis* with favorable results [16]. Quinolones (ciprofloxacin or moxifloxacin) or tetracyclines (minocyline) are active against *M. hominis* and moxifloxacin appears to have the greatest activity as the most effective therapeutic agent. If *M. hominis* was correctly identified as underlying pathogen, the antibiotics would be therefore switched to the right agents with a marked improvement of clinical syndromes and a favorable result [16, 19]. The patient in our case had the regimen of the combination of moxifloxacin and doxycycline and had positive response.

In summary, the postoperative infection after hip replacement secondary to *M. hominis* is rare. Currently there are 4 published reports of septic arthritis caused by *M. hominis* after hip or knee replacement in adults. Our case added to this body of evidence. The clinicians should recognize the possibility of *M. hominis* involvement in postoperative infections without microbiological findings or response to standard therapy, and consider changing antibiotic regimen.

## Abbreviations

CRP: C-reactive protein (CRP)
ESR: Erythrocyte sedimentation rate
WBC: White blood cell

## References

[1] Allen-Daniels MJ, Serrano MG, Pflugner LP, Fettweis JM, Prestosa MA, Koparde VN, Brooks JP, Strauss JF, Romero R, Chaiworapongsa T (2015). Identification of a gene in Mycoplasma hominis associated with preterm birth and microbial burden in intraamniotic infection. Am J Obstet Gynecol.

[2] Flouzat-Lachaniette CH, Guidon J, Allain J, Poignard A (2013). An uncommon case of mycoplasma hominis infection after total disc replacement. Eur Spine J.

[3] Phuah CL, Javid B, Aliyu SH, Lever AM (2007). A case of mycoplasma hominis septic arthritis postpartum. J Infect.

[4] Krijnen MR, Hekker T, Algra J, Wuisman PI, Van Royen BJ (2006). Mycoplasma hominis deep wound infection after neuromuscular scoliosis surgery: the use of real-time polymerase chain reaction (PCR). Eur Spine J.

[5] Zhou M, Wang P, Chen S, Du B, Du J, Wang F, Xiao M, Kong F, Xu Y (2016). Meningitis in a Chinese adult patient caused by mycoplasma hominis: a rare infection and literature review. BMC Infect Dis.

[6] Whitson WJ, Ball PA, Lollis SS, Balkman JD, Bauer DF (2014). Postoperative mycoplasma hominis infections after neurosurgical intervention. J Neurosurg Pediatr.

[7] Yokoyama H, Domen T, Hiragata S, Ogawa T, Matsumoto T, Ishizuka O (2016). Postoperative mycoplasma hominis infection after robot-assisted laparoscopic radical prostatectomy: a case report. Asian J Endosc Surg.

[8] Bergin SM, Mendis SM, Young B, Binti Izharuddin E. Postoperative mycoplasma hominis brain abscess: keep it in mind!. BMJ Case Rep. 2017;2017:1–3. 10.1136/bcr-2016-218022 PMC5255544 28069785

[9] Le Guern R, Loiez C, Loobuyck V, Rousse N, Courcol R, Wallet F (2015). A new case of mycoplasma hominis mediastinitis and sternal osteitis after cardiac surgery. Int J Infect Dis.

[10] Meyer RD, Clough W (1993). Extragenital mycoplasma hominis infections in adults: emphasis on immunosuppression. Clin Infect Dis.

[11] Miranda C, Camacho E, Reina G, Turino J, Rodriguez-Granger J, Yeste R, Bautista MF, Garcia M, Alados JC, De la Rosa M (2005). Isolation of mycoplasma hominis from extragenital cultures. Eur J Clin Microbiol Infect Dis.

[12] Fernandez S, Nicolas D, Pericas JM, Castro Rebollo P, Vila J, Miro JM, Blanco JL, Nicolas JM (2017). A case of Mycoplasma hominis disseminated infection in a human immunodeficiency virus-1-infected pregnant woman with hypogammaglobulinemia. J Microbiol Immunol Infect.

[13] Romeu Prieto JM, Lizcano Lizcano AM, Lopez de Toro Martin Consuegra I, Largo Pau J, Lopez Almodovar LF, Garcia Camacho E (2015). Culture-negative endocarditis: mycoplasma hominis infection. Rev Esp Cardiol.

[14] Lee EH, Winter HL, van Dijl JM, Metzemaekers JD, Arends JP (2012). Diagnosis and antimicrobial therapy of mycoplasma hominis meningitis in adults. Int J Med Microbiol.

[15] Wylam ME, Kennedy CC, Hernandez NM, Peters SG, Maleszewski JJ, Cassivi SD, Scott JP (2013). Fatal hyperammonaemia caused by mycoplasma hominis. Lancet.

[16] Qiu HJ, Lu WP, Li M, Wang ZM, Du QY, Wang AM, Xiong Y (2017). The infection of Mycoplasma hominis after total knee replacement: Case report and literature review. Chin J Traumatol.

[17] Lee JH, Lee JH, Lee NY, Ha CW, Chung DR, Peck KR (2009). Two cases of septic arthritis by mycoplasma hominis after total knee replacement arthroplasty. Korean J Lab Med.

[18] Sneller M, Wellborne F, Barile MF, Plotz P (1986). Prosthetic joint infection with mycoplasma hominis. J Infect Dis.

[19] MacKenzie CR, Nischik N, Kram R, Krauspe R, Jager M, Henrich B (2010). Fatal outcome of a disseminated dual infection with drug-resistant mycoplasma hominis and Ureaplasma parvum originating from a septic arthritis in an immunocompromised patient. Int J Infect Dis.

[20] Pailhories H, Rabier V, Eveillard M, Mahaza C, Joly-Guillou ML, Chennebault JM, Kempf M, Lemarie C (2014). A case report of mycoplasma hominis brain abscess identified by MALDI-TOF mass spectrometry. Int J Infect Dis.

[21] Cuchý E, Cherta I, Garau J (2000). Mycoplasma hominis catheter-related infection in a patient with multiple trauma. Clin Microbiol Infect.

[22] Reissier S, Masson R, Guerin F, Viquesnel G, Petitjean-Lecherbonnier J, Pereyre S, Cattoir V, Isnard C (2016). Fatal nosocomial meningitis caused by mycoplasma hominis in an adult patient: case report and review of the literature. Int J Infect Dis.

[23] Koshiba H, Koshiba A, Daimon Y, Noguchi T, Iwasaku K, Kitawaki J (2011). Hematoma and abscess formation caused by mycoplasma hominis following cesarean section. Int J Women's Health.

[24] Tyner HL, Virk A, Nassr A, Razonable R (2016). Mycoplasma hominis vertebral spine infection: case report and a review of infections of bone and joints. J Infect Chemother.

